# Bone mass in schizophrenia and normal populations across different decades of life

**DOI:** 10.1186/1471-2474-10-1

**Published:** 2009-01-01

**Authors:** Jenn-Huei Renn, Nan-Ping Yang, Ching-Mo Chueh, Chih-Yuan Lin, Tsuo-Hung Lan, Pesus Chou

**Affiliations:** 1 Community Medicine Research Center and Institute of Public Health, National Yang-Ming University, Taipei, ROC; 2 Yuli Veterans Hospital, Veterans' Affairs Commission, Executive Yuan, Hualien, Taiwan, ROC; 3 Department of Geriatrics and Orthopedic Surgery, Tao-Yuan General Hospital, DOH, Executive Yuan, Tao-Yuan, Taiwan, ROC; 4 Department of Psychiatry, Kuang Tien General Hospital, Taichung, Taiwan, ROC; 5 Department of Psychiatry, Yang-Ming University, Taipei, Taiwan; 6 Department of Psychiatry, Taichung Veterans General Hospital, Taichung, Taiwan; 7 Division of Mental Health & Substance Abuse, National Health Research Institute, Zhunan, Taiwan

## Abstract

**Background:**

Chronic schizophrenic patients have been reported as having higher osteoporosis prevalence. Survey the bone mass among schizophrenic patients and compare with that of the local community population and reported data of the same country to figure out the distribution of bone mass among schizophrenic patients.

**Methods:**

965 schizophrenic patients aged 20 years and over in Yuli Veterans Hospital and 405 members aged 20 and over of the community living in the same town as the institute received bone mass examination by a heel qualitative ultrasound (QUS) device. Bone mass distribution was stratified to analyzed and compared with community population.

**Results:**

Schizophrenic patients have lower bone mass while they are young. But aging effect on bone mass cannot be seen. Accelerated bone mass loss during menopausal transition was not observed in the female schizophrenic patients as in the subjects of the community female population.

**Conclusion:**

Schizophrenic patients have lower bone mass than community population since they are young. Further study to investigate the pathophysiological process is necessary to delay or avoid the lower bone mass in schizophrenia patients.

## Background

Osteoporosis is a bone disease that can reduce both bone mass and bone strength. It is typically thought to be age-related [1] and can cause serious bone fractures that can have significant and even devastating physical, psychological and financial consequences for patients and their families [2-4]. The prevalence of schizophrenia is about 1% worldwide. Rigotti and colleagues[5] first reported decreased bone density in patients with a confirmed mental disorder as anorexia nervosa. Other researchers have reported similar findings in anorexia [6-8]. Osteoporosis was also reported in schizophrenia[9,10] patient and the prevalence of non-traumatic fractures in chronic schizophrenic patients has been reported to be about 25% in a cross-sectional survey[10]. A prevalence of osteoporosis in chronic schizophrenic patients has been reported, but a study of the prevalence of osteoporosis in chronic schizophrenic patients on a large population has not previously been reported. In this study, 965 chronic in-patient schizophrenic cases in Yuli Veterans hospital of Yuli town in Taiwan were surveyed; 405 members of the community population were also surveyed as the control group simultaneously. The bone mass distribution in the chronic schizophrenic patients was evaluated and compared with that of the community population subjects.

## Methods

### Study Population

The present study included 965 patients, who were older than 20 years old, chronic schizophrenia admitted to the Psychiatric Department of Yuli Veterans Hospital in Taiwan, ROC, between 2003 and 2004. All of these patients met the DSM-IV criteria for schizophrenia as diagnosed by psychiatrists in Yuli Veterans Hospital. Patients with severe extra-pyramidal symptoms (EPS), poor disease control, or severe debility that precluded cooperation with the bone density survey were excluded from the study. For comparison, 405 community members, who were older than 20 years old, living in the same district as Yuli Veterans Hospital, also entered the study. All residents who lived in this district were visited at their home. Those who were not living in Yuli Town during the survey period or did not give consent or had medical or mental disability that prevented them from completing the examination were excluded. The study was approved by the medical ethics policy of the Institutional Review Board Committee at Yuli Veterans Hospital (serial No. 92-11-02A).

### Bone Mass and Quantitative Ultrasound

A QUS-II Calcaneal Ultrasonometer (Metra Biosystems, Mountain View, CA, USA) was used to measure bone density as broadband ultrasound attenuation (BUA) data. Ultrasound bone densiometry is reported to correlate well with the results of DXA and can predict osteoporosis-related fracture[11,12] or detect bone fragility [13-15]. But there are many authors who question the precision of peripheral bone mineral density. Some authors reported that QUS parameters couldn't be used to predict osteopenia and that the sensitivities and specificities of QUS parameters were not high enough to be used as an alternative method of dual-energy x-ray absorptiometry (DXA) [16-19]. DXA is gold standard for measuring BMD is currently[15,20,21]. However, DXA scans are time-consuming, costly and expose the patient to radiation, which makes it a less-than-ideal method for a large population survey. On the contrary, a calcaneal ultrasonometer is timesaving, portable and suitable method for large surveys. For this reason, we chose to use it in this study.

Osteoporosis is defined by decreased bone mineral density (BMD) to a level of or less than -2.5 SD of the mean value of young adults. Firstly, our group assessed the precision of the quantitative ultrasound densitometry. Intra-test precision was calculated from three repeated scans with repositioning in 25 volunteers; the short-term coefficient of variation was 3.5% for BUA. T-scores used in our data were calculated from measured BUA data, the apparatus' specific threshold and further adjustment using data from Chinese patients living in Taiwan as reported by N.P. Yang[22]. Low bone mass was defined by a T-score of less than -1.0, and severe low bone mass was defined by a T-score of -2.5 or lower in this study. Weight and height data were also measured and collected.

### Statistical Analysis

All subjects were classified into two subject groups. Admitted schizophrenic patients were classified as schizophrenic patient group. Residents lived in the community were classified as community population group. All the subjects were classified into 5 age groups in decades of age from 20 to 60 years old and 60 years above. Correlation between measured BUA value and weight, gender, subject groups, and age groups was tested by fractional factorial generalized linear model. Interactions between age groups and subject groups were also tested in the same model. Effects of weight, gender and age groups on the differences of means of measured BUA value was tested with generalized linear model in each subject group. Trends of mean BUA values between age groups stratified by subject groups and gender was tested by nonparametric test for trend. Prevalence of osteoporosis data were stratified by both gender and age groups for both groups and reported by descriptive analysis and was compared further with the prevalence reported in Taiwan. All statistical calculations were conducted using the Stata 10/SE system for Mac.

## Results

The demographic data and BUA value results stratified by subject groups, age groups and gender are presented in Table 1. There were 623 (65%) male and 342 (35%) female patients in the schizophrenic patient group. Mean age was 47.6 ± 15.9 (range 21–95) years in the male patients and 46.8 ± 11.2 (range 20–78) years in the female patients. In the community population group, there were 183 (45%) male and 222 (55%) female subjects; mean age was 60.0 ± 18.2 (range 20–89) years in male and 54.2 ± 16.0 (range 20–88) years in the female.

**Table 1:** The Basic Characteristics of the Schizophrenic Patients compared to community population, sampled from the same community, Yu-Li area, Taiwan

Age Strata (y/o)	Studied Subjects		BUA (dB/MHz)		Weight (Kgw)	
	Schizophrenic	Community	Schizophrenic	Community	Schizophrenic	Community
	No.(%)	No.(%)	Mean (S.D.)	Mean (S.D.)	Mean (S.D.)	Mean (S.D.)
Male						
20–29	59(9.5)	12(6.6)	77.1(15.3)	86.1(21.6)	69.3(13.9)	63.9(8.9)
30–39	184(29.5)	18(9.8)	82.3(17.0)	90.1(17.2)	71.3(19.2)	71.3(10.5)
40–49	177(28.4)	30(16.4)	83.9(18.6)	92.0(19.4)	67.2(15.3)	69.5(10.9)
50–59	77(12.4)	28(15.3)	80.3(18.2)	83.7(20.7)	67.4(13.4)	67.3(8.3)
60 or more	126(20.2)	95(51.9)	82.2(18.2)	78.0(21.1)	52.9(9.2)	66.8(10.9)
Total	623	183				
						
Female						
20–29	22(6.4)	16(7.2)	71.9(26.5)	85.3(11.1)	69.6(12.3)	53.7(7.7)
30–39	84(24.6)	33(14.9)	76.9(20.8)	85.8(11.7)	70.2(15.6)	57.3(9.9)
40–49	97(28.4)	38(17.1)	81.2(20.5)	86.3(16.9)	71.9(19.1)	59.8(10.7)
50–59	97(28.4)	45(20.3)	82.3(20.1)	72.0(16.5)	68.4(7.8)	58.0(9.2)
60 or more	42(12.3)	90(40.5)	79.0(19.9)	68.7(17.1)	65.3(6.2)	57.5(10.2)
Total	342	222				

**Totally**	965	405				

**Figure 1:**
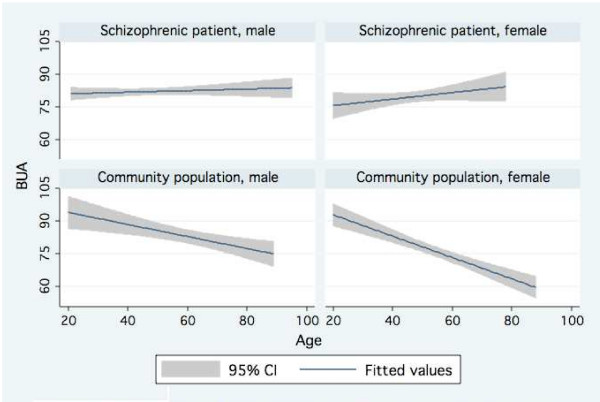
**BUA vs. age with 95% confidence and fit line by subject population and sex**.

Mean BUA values were lower for male schizophrenic patients as compared with male community population group of same age groups if they were 60 years old or younger. But mean BUA value was higher for male schizophrenic patients in the age group older than 60 years. Mean BUA values were also lower for female schizophrenic patients as compared with female community population group of same age groups if they were younger than 50 years old. But mean BUA values for female schizophrenic patients were higher in age groups older than 50 years. This is demonstrated in Fig. 1. A significant trend of mean BUA value with aging was observed in males (p = 0.001) and females (p < 0.001) of community population. It is insignificant in male and female schizophrenic patients group.

The male schizophrenic patient group has a higher mean weight than the male community population group except in the age group 40–50 and the age group > 60 years old. The mean weight of the female schizophrenic patient group is higher than the female community population group across all age groups.

Table 2 shows the fractional factorial generalized linear model result of subject groups, gender, weight, and age groups on BUA. The BUA value of females is significant lower than that of males (p < 0.001). Lean subjects have a significantly lower BUA value (p < 0.001). The schizophrenic patient group has a significantly lower BUA value (p < 0.01). Significant effect is seen in the age groups 20–29 (p < 0.001), 30–39 (p < 0.01) and 40–49 (p < 0.05) years old on BUA value. Significant interaction can be seen between subject groups and age group 50–59 and age group > 60 years old.

**Table 2:** Comparison of BUA in schizophrenic patient and community population group stratified by age group, gender by fractional factorial generalized linear model

	(dependent variable = BUA)		
Independent variables	coef.	std. err.	p-value
Gender (female vs. male)	-4.251	1.054	***
Weight (kgw)	0.158	0.036	***
Group^#^(community vs. schizophrenic)	13.069	4.096	**
Age group 20–39	-23.949	6.497	***
Age group 30–39	-15.424	4.980	**
Age group 40–49	-10.727	4.777	*
Age group 50–59	-----	-----	n.s.
Age group ≧ 60	-----	-----	n.s.
Group*Age group 20–39	-----	-----	n.s.
Group*Age group 30–39	-----	-----	n.s.
Group*Age group 40–49	-----	-----	n.s.
Group*Age group 50–59	-16.723	4.826	**
Group*Age group ≧ 60	-20.967	4.563	***
Constant	80.987	4.741	***

n.s. : not significant

* : p < 0.05

** : p < 0.01

*** : p < 0.001

#: schizophrenic patient group and community population group

Generalized linear model was used to test the effect of gender, weight, and age on the difference of mean BUA value and was stratified by subject groups. The result is shown in Table 3. Males have significantly higher mean BUA value than females in both schizophrenic patient and community population groups (p < 0.05). Higher BUA value can be significantly seen in heavier subjects in both subject groups (p < 0.05 in schizophrenic patient group, and p < 0.001 in community population group). In the community population group, the coefficient of age is negative and is significant (p < 0.001). But the coefficient of age is positive and is significant also (p < 0.05) in the schizophrenia patient group. Interaction is tested also between age and weight and is insignificant.

**Table 3:** Comparison of BUA by gender, weight, and age in schizophrenic patient and community population group with generalized linear model

	Schizophrenic patient			Community population		
Independent variable	β	std. err.	p-value	β	std. err.	p-value
Gender (male vs female)	-2.731	1.278	*	-4.474	1.986	*
Weight (kgw)	0.105	0.041	*	0.445	0.086	***
Age (years)	0.097	0.044	*	-0.362	0.052	***
Constant	73.189	4.218	***	78.992	8.206	***

* : p<0.05

** : p<0.01

*** : p<0.001

Table 4 shows the comparison of prevalence of low bone mass (t-score <-1 computed from BUA value) and severe low bone mass (t-score ≦ -2.5 computed from BUA value) between schizophrenic patient group, community population group and surveyed data in Kinmen, Taiwan[22]. The prevalence of low bone mass and severe low bone mass is both higher in schizophrenic patients in age group 20–29 and age 30–39 years old. In age groups 40–49, 50–59 and > 60 years old, the prevalence of low bone mass and severe low bone mass of schizophrenic is also higher than the community population group and the surveyed data in Kinmen, Taiwan.

**Table 4:** Comparison of low bone mass condition between the comminuty population and a schizophrenic population in Taiwanese

	prevalence of low bone mass [t < 1.0]			prevalence of severe low bone mass [t ≦ -2.5]		
	Schizophrenic population in Yuli area	Community population in Yuli area	Community-surveyed People in Kinmen, Taiwan, 2000–2003§	Schizophrenic population in Yuli area	Community people in Yuli area	Community-surveyed People in Kinmen, Taiwan, 2000–2003§
	(%)	(%)	(%)	(%)	(%)	(%)
Age Strata						
Male						
20–29 y/o	54.2	50.0	-----	15.3	0	-----
30–39 y/o	42.9	33.3	-----	9.8	0	-----
40–49 y/o	37.3	33.3	35.5	6.8	3.3	3.9
50–59 y/o	46.8	50.0	42.5	13.0	10.7	7.1
> 60 y/o	48.4	55.8	55.8	6.4	25.3	16.9
Female						
20–29 y/o	77.3	18.8	-----	50.0	0	-----
30–39 y/o	50.0	18.2	-----	26.2	0	-----
40–49 y/o	42.3	31.6	38.4	17.5	5.3	6.7
50–59 y/o	46.4	68.9	52.9	14.4	28.9	16.5
> 60 y/o	52.4	78.9	84.1	19.1	31.1	50.9

Note: The above three population were screened for low bone mass using the same ultrasonography, QUS-II. The t-score was calculated using machine BUA value and adjusted as: (BUA_individual _– 92.72)/13.36 for the male, and (BUA_individual _– 87.90)/10.68 for the female. Those were based on the published reference§.

§Nan-Ping Yang, Shao-Yuan Chuang, Shui-Hu Chen, Pesus Chou. Screening for low bone mass with quantitative ultrasonography in a community without dual X-ray absorptiometry: population-based study. BMC Musculoskeletal Disorders 2006;7:24.

## Discussion

The results of our study reveal lower BUA value in the schizophrenic patient group while these patients are young. The prevalence of low bone mass and severe low bone mass showed similar changes when compared with community populations from the same geographical area and from Kinmen Island, which is in a different geographical location. The geography- related effect can be ruled out in this study. Yuli town is in a rural area in the east part of Taiwan. Healthier young people move to the cities for work. For this reason, the sample size of the 20–29 and the 30–39 year old group is small in the community population group. This is also the reason of higher osteoporosis prevalence in the 20–29 year-old male group.

Poorer bone mass in psychiatric patients has been reported in many literatures[9,10,23]. Since it can be seen even in the age group 20–29 years, inadequate peak bone mass build or accelerated bone absorption is the possible cause of low bone mass. Reported risk factors for the increased prevalence of osteoporosis in psychiatric patients include polydipsia[24], the use of neuroleptics [9,25-27] and the resulting hyperprolactinemia[11,28-32], heavy smoking[33], poor diet, drug and alcohol abuse[34], and lack of exercise[30]. These factors were supposed to accelerate bone absorption. The onset of schizophrenia typically occurs during adolescence and young adulthood. Therefore, the use of antipsychotic medications with their attendant metabolic changes also begins at the same stage. Lifestyle changes also begin at about the same period. Each of these factors or a combined effect of these factors may inhibit the build-up of peak bone mass or accelerate bone loss, and may be the reason for low BUA values in age groups younger than 50 years.

A rapid decrease of bone mass and an increase of osteoporosis during the menopausal transition are reported in the community female population[35,36]. However, the trend test of BUA value in female schizophrenic patients cannot show the transition effect in our study. Hyperprolactinemia caused by the medication of many kinds of neuroleptics was suspected to suppress gonadotropins and gonadal hormones[37,38]. Low BMD values and hypogonadism was reported in young schizophrenic women with hyperprolactinemia who were treated with prolactin-raising antipsychotics[39]. Effect of decrease of gonadotropin on bone mass during menopausal transition in female schizophrenic patients may be irrelevant for female subjects in the community population. Further study is necessary to explain this.

The BUA value keeps relatively stable while aging, as shown in trend analysis. In the generalized linear model, BUA increased significantly with age increase. Aging is not a risk factor of poor bone mass in schizophrenic patient group in our study, and is a protective factor as the statistical result. Aging has been reported to be a risk factor of osteopenia in psychiatric patients treated with prolactin-elevating antipsychotics.[40] Our study does not include antipsychotics used to treat these patients. Classic neuroleptics were reported to cause hyperprolactinemia and an increased prevalence of osteoporosis than atypical neuroleptics [36,37,41,42]. However, some atypical neuroleptic agents are reported to produce either the same or more pronounced hyperprolactinemia[43,44]. Interaction between aging and prolactin-elevating antipsychotics may exist. Study is necessary to explore the effect of aging on schizophrenic patients and to explore whether aging is a protective factor.

The chronic schizophrenic patient may also have several of the risk factors for osteoporosis-related fractures, commonly including unsteady gait, decreased physical activity and the use of sedative medications and low cognitive function. Residents of a long-term care unit are at a higher risk of osteoporotic fractures due to one or more of following risk factors: advanced age, poor physical function, low muscle strength, decreased cognition, dementia, poor nutrition and the use of certain medications.[4] The process of accelerated osteoporosis in schizophrenic patients needs to be explored and may be prevented or changed to preserve bone mass in these patients.

## Conclusion

In this study, schizophrenic patients have lower BUA values since they are young when compared with the community population. Aging and menopausal transition effect on bone mass in the general female population cannot be seen in the schizophrenic patient group. The bone mass distribution of schizophrenic patients is different from that of the community population. This is a cross-sectional study, and does not include the information of sex hormone, bone metabolism related hormones, the type of antipsychotic medication used and bone turnover markers is its limitation. Use of ultrasonographic assessments instead of DXA scans and the assessment of bone mass at only one location is another limitation of this survey. Further studies are necessary to clarify the hormonal changes, bony metabolism and effects of medication on bone mass characteristics in schizophrenic patients. Prospective study begins from the onset of schizophrenia may be also necessary. Once the cause and effect relationships are clearer, intervention may be used to either prevent or delay the onset of osteoporosis in these patients.

## Competing interests

The whole study is funded by research budget of Yuli Veterans Hospital for supporting and encouraging staff to perform study related to medicine. Otherwise, there are no other competing interests.

## Authors' contributions

JHR conducted and performed the whole project. NPY carried the data analysis. CMC and CYL confirmed the schizophrenia diagnosis. THL sampled the schizophrenic patients within all inpatient in Yuli Veterans Hospital and arranged and recorded result and arranged the schedule of ultrasound densitometer. PC designed and supervised the whole project.

## Pre-publication history

The pre-publication history for this paper can be accessed here:


